# Enucleation of the Eye

**Published:** 1861-12

**Authors:** E. L. Holmes

**Affiliations:** Chicago, Ills.


### CHICAGO
### MEDICAL JOURNAL.

VOL. IV.] DECEMBER, 1861. [No. 12.

### ORIGINAL COMMUNICATIONS.

### "ENUCLEATION OF THE EYE."

By E. L. HOLMES, M. D., of Chicago, Ills.

"Conservative Surgery" in all its relations to the science of medicine claims the most earnest attention of our profession, since it is much more in accordance with an enlightened humanity and more creditable to the surgeon to save a diseased or an injured organ, than to destroy it, even in the performance of a brilliant operation.

For special reasons the destruction of an organ, so important as the eye, is a measure in surgical practice which should only be adopted after careful deliberation and in the presence of absolute necessity.

The loss of the globe of a single eye, even when its powers of vision are irretrievably destroyed, is by no means a trifling misfortune. However reconciled the patient may become to his deformity, it is often difficult to conceal it from those with whom he is in daily contact. The deformity is often so repulsive to many individuals, that they dislike to give employment to unfortunate patients thus afflicted. Although an artificial eye in many instances almost perfectly conceals the mutilation from ordinary observation, it must be remembered that the expense of an eye often places it beyond the means of the patient; it is exceedingly fragile and very liable to be broken, especially when worn by laborers and domestics; moreover, the natural secretions of the eye and the constant friction of the lids roughen the surface of the enamel in a few years, which irritates the mucous membrane of the lids and necessitates the purchase of a new eye.

We have known several poor patients who had saved sufficient means, after considerable exertion, to purchase an artificial eye, which they broke in less than a month after. Their deformity was a serious obstacle to their efforts to obtain employment.

It is a matter of considerable importance, therefore, to the patient to preserve an affected eye with as little deformity as possible, even though it be entirely useless as regards vision. And yet there are affections of the eye constantly falling under the care of the opthalmic surgeon, in which the comfort and health of the patient, and even the preservation of the vision of the other eye, demand the extirpation of the eye first diseased.

In treating these cases, practitioners seem usually to err in endeavoring too long to avoid an operation rather than in urging it upon patients, when not required.

It is our present purpose to consider briefly these cases and to show under what circumstances our best authorities advise the total extirpation of the eye.

We shall not discuss the propriety of removing the eye when affected with malignant diseases, since this involves points in pathology and practice independent of the principles now under consideration.

There are two classes of opthalmic diseases in which the question of extirpation of the eye usually arises: one including the different degrees of staphyloma, the other embracing chronic inflammation of the deep tissues, especially the iris and choroid. In the first class the operation is usually performed for the purpose of removing a deformity, and in the second, to relieve pain and prevent sympathetic ophthalmitis

—one of the gravest affections of the eye, the surgeon is called upon to treat. In simple staphyloma of the cornea, when the diseases which caused it have passed away and the balance of the eye is free from inflammation, the amputation of the cornea and iris alone is usually sufficient. The wound generally heals without trouble, leaving a good "stump" for an artificial eye.

Two accidents may occur in performing this simple operation, which may place the case in the second category just mentioned: Either the wound may not heal kindly and a slow form of inflammation invade the remainder of the eye, causing neuralgic pain and irritation; or hæmorrhage may take place behind the vitreous humor from the delicate vessels of the retina and choroid, which sometimes become ruptured by the removal of the pressure from them, when the natural fluids of the eye are evacuated. Inflammation and quite extensive suppuration usually follow. In either of these accidents the remaining portion of the eye should be removed to relieve the pain and to ensure safety to the other eye.

There seems to be little uncertainty expressed by ophthalmologists in regard to the treatment most suitable in the second class of cases. Observation has shown that sympathetic ophthalmitis is almost wholly confined to those diseases and injuries which produce chronic inflammation of the deep tissues of the eye, which is too often found difficult to relieve by any treatment except the total extirpation of the eye—or "enucleation," as it is now sometimes called.

Although any affection, which produces chronic inflammation of the choroid and iris in one eye, particularly where the iris becomes extensively attached to the lens, may, if not relieved by treatment, cause the sympathetic affection in the other, it is found that four conditions are especially liable to cause it:

1st. Where foreign bodies are lodged in the globe.

2d. Where a dislocated lens becomes a source of irritation to the eye.

3rd. Where punctured injuries of the eye do not heal readily, but leave deep seated inflammation of the eye.

4th. Injuries, in which large coagula are formed within the globe.

These and similar affections are generally accompanied with neuralgic pain in the eye and over the brow and temple, and finally cause atrophy of the globe.

It must not be supposed, however, that these affections, even the gravest, invariably terminate in sympathetic ophthalmitis. Many patients escape after protracted suffering with loss of but one eye. Still it is the duty of the surgeon, in view of the terrible consequences attending the sympathetic disease, to advise the immediate removal of the affected eye. We have known six cases of injury of one eye, followed by loss of the other from this serious disease. There is not the least doubt in our mind, that the extirpation of the eye first affected would not only have saved the other, but also spared the patients weeks and months of excruciating pain.

There is no need of dwelling upon the description of the symptoms and course of sympathetic ophthalmitis, since the subject is discussed at length in our standard works upon diseases of the eye. We will merely state that the disease commences in the second eye, at a period varying in length from a few weeks to several months subsequent to the injury of the first. Vision usually becomes dim in the early stage of the disease, and finally becomes extinct. There is a peculiar pain and redness in the globe, indicative of inflammation of the deep structures of the eye, which, as we have already stated, almost invariably proves rebellious to any constitutional or local treatment. It was not till quite recently that the only reliable preventive treatment has been found to consist in extirpating the eye primarily affected, before the unpleasant symptoms appear in the sound eye.

The operation, recommended by Bonnet, Critchett and other well known opthalmic surgeons, consists: 1st. In dividing the conjunctiva to the sclerotica by a circular incision parallel with the circumference of the cornea; 2d. In cutting the recti muscles close to the sclerotica, as in operating for strabismus; 3d. In dividing the optic nerve as near the globe as possible, by means of a pair of curved blunt pointed scissors, passed behind the eye, and 4th. In cutting the oblique muscles as the eye is raised from the orbit.

If the eye is firmly held by means of a suitable pair of toothed forceps, the eye can be thus "enucleated" with great ease and rapidity. There is usually but little hæmorrhage or pain after the operation. There may sometimes be slight œdema of the lids for a few days, but we have never seen anything happen to prevent patients from sitting up, and even in going about the house the next day. In a couple of weeks the wound will generally be entirely healed.

Wet compresses are the only dressing required.

Of late we are in the habit of injecting cool water freely into the orbital cavity immediately after the operation, to remove the coagula and prevent their formation. There is then less unpleasant discharge during the progress of cicatrization.

The conjunctiva, muscles and the lymph, which becomes organized in the formation of the cicatrix, furnish a good support for an artificial eye which, if it does not enable the eye to move as freely as a larger "stump," is less liable to become irritated and inflamed by the presence of the eye.

This subject is an important one; prognosis in any case as to the effects which an injury or disease will have upon the other eye, is always uncertain; it is worthy of consideration, whether it is not better, when vision in one eye has become extinct, and the inflammation of the tissues has continued a long time in spite of treatment to extirpate the eye, in a dozen cases, when the other eye might possibly have escaped, than to suffer a single patient to become totally blind by too long a delay!

We would refer our readers to recent English and other foreign authorities, and also to papers in vol. VI (1859) of the New York *Journal of Medicine*, by Drs. Agnew and Bumstead, of the New York Eye Infirmary.

